# Association Between Deforestation and the Incidence of Snakebites in South Korea

**DOI:** 10.3390/ani15020198

**Published:** 2025-01-13

**Authors:** Seoheui Lee, Junyeong Lee, Kyung-Duk Min

**Affiliations:** 1 Department of Big Data, Chungbuk National University, Cheongju 28644, Chungbuk, Republic of Korea; 2 Department of Management Information Systems, Chungbuk National University, Cheongju 28644, Chungbuk, Republic of Korea; 3 College of Veterinary Medicine, Chungbuk National University, Cheongju 28644, Chungbuk, Republic of Korea

**Keywords:** snakebite, conservation, natural resource, risk factor, logistic model, spatial analysis

## Abstract

Snakebites are a significant global health concern; despite various known risk factors, the role of environmental changes, such as deforestation, remains underexplored. Therefore, we investigated the association between deforestation and snakebite incidence in South Korea. Aggregated data were obtained from the National Health Insurance Service (NHIS) and the National Emergency Department Information System (NEDIS) from 2014 to 2021. The main explanatory variable was the level of deforestation by district and year. Additional covariates included altitude, average temperature, population size, proportion of urban land cover, proportion of agricultural land cover, and level of ecological preservation. Twelve statistical models were employed to assess the association between deforestation and snakebite incidence. The odds ratios and relative risks ranged between 1.217 and 1.452 and between 1.078 and 1.175, indicating a significant positive association between these two factors. These findings suggest that deforestation notably increases snakebite risk; collectively, our findings can help develop targeted preventive measures and healthcare strategies to reduce snakebite risk globally.

## Simple Summary

Snakebites are a serious public health problem worldwide. Various risk factors affect the incidence of snakebites, Previous studies on deforestation affecting the incidence of snakebites are insufficient. Thus, the present study investigated the association between deforestation and snakebites in South Korea. To prove the association among these factors, we conducted twelve statistical models. As a result, the odds ratios and relative risks ranged between 1.217 and 1.452 and between 1.078 and 1.175. This study proved that snakebites increase significantly as deforestation increases. These findings can help establish a system and policy that decreases the risk of snakebites.

## 1. Introduction

Snakebites are a serious global public health concern. Annually, an estimated 4.5–5.4 million snakebite accidents occur worldwide [1], resulting in approximately 400,000 disabilities and approximately 100,000 fatalities [2]. In America, the annual incidence stands at 57,500 snake bites, leading to approximately 370 deaths [3]. Sub-Saharan African countries report over 300,000 snakebites annually [4], whereas Pakistan records approximately 40,000, with approximately 8200 fatalities [5]. In Nepal, over 20,000 envenoming cases occur each year, with approximately 1000 deaths, and in Sri Lanka, approximately 33,000 envenomed snakebites occur annually [1]. The national annual costs of managing snakebites, including medical expenses, range from US$126,319 in Burkina Faso to US$13,802,550 in Sri Lanka [1]. The high medical expenses in Sri Lanka related to high snakebite incidence are estimated at over $10 million annually.

Various factors, including climatic conditions, demographic characteristics, and socioeconomic aspects, influence snakebite incidence and serve as explanatory variables. Among climatic factors, precipitation is particularly significant; while many snake species prefer warm environments, certain species thrive in cooler, more humid conditions. Increased rainfall can enhance their activity and positively impact their breeding [6]. Additionally, research has shown that snakes in various regions often become more active following rainfall due to the availability of food and suitable microhabitats [7]. This increased activity leads to more frequent encounters between humans and snakes, resulting in a higher incidence of snakebites [7]. Colombia is categorized as both tropical and subtropical, with the northern regions experiencing a tropical climate characterized by warm and humid conditions, while the central areas exhibit a subtropical climate with mild temperatures. The rainy season in Colombia is particularly significant for snake activity as increased rainfall during this time enhances their movement and breeding, leading to more frequent encounters between humans and snakes. Demographically, snakebite incidence in India is higher among men than women, primarily owing to societal gender roles. Men, often engaged in outdoor work, particularly in agriculture, encounter snake-prone environments more frequently, thereby increasing their risk of snakebites [8].

Deforestation could be another potential risk factor for snakebites, as habitat destruction reduces available environments for primary animal habitats [9]. Snakes favor areas with tree roots, shrubs, and rocks, and habitat destruction compromises their survival. Moreover, deforestation causes a decrease in the availability of small mammals, birds, and amphibians, which are key prey for snakes [10]. While it is true that snakes often migrate in search of more suitable habitats, including areas with greater vegetation cover, recent research suggests that urban environments are increasingly becoming attractive to certain snake species. This is particularly evident in regions where natural habitats are being destroyed or degraded due to deforestation and urbanization. Urban areas can provide adequate shelter and food sources, such as rodents, which thrive in human settlements. For example, heat islands in cities create warmer microclimates, which can appeal to snakes seeking optimal temperature conditions for activity and reproduction. Furthermore, as snakes lose their natural habitats, they are compelled to venture into densely populated areas, increasing the likelihood of human–snake encounters. This is supported by studies that show higher snakebite incidents in areas undergoing rapid urban expansion. Therefore, while migration to vegetative areas is common, urban areas can also offer conditions favorable for certain species due to food availability, shelter, and climate, leading to increased human–snake interactions. However, examining deforestation as a potential risk factor for snakebites is relatively uncommon.

To address these gaps in the literature, we aimed to investigate the association between deforestation and snakebite incidence using empirical data from South Korea. By leveraging high-quality data from a country recognized for its advanced medical infrastructure, including a robust digital health system and effective emergency response capabilities, we ensured a reliable and valid analysis. In contrast, many developing countries often struggle with incomplete data collection due to underdeveloped health systems and inadequate emergency response resources. Furthermore, we aimed to identify potential risk factors for snakebites to gain insights for the development of effective strategies and policies aimed at reducing snakebite incidences.

## 2. Materials and Methods

### 2.1. Study Design

An ecological study was conducted using aggregated data to examine the association between deforestation and the incidence of snakebites. Both outcome and explanatory variables, including the number of snakebite cases, deforestation levels, and other covariates (altitude, temperature, population, proportion of urban area, proportion of agricultural area, proportion of protected area and proportion of forested area), were obtained at the district level. All 250 districts in South Korea were included based on data availability, and the study period was from 2014 to 2021. This resulted in 2000 study units (250 districts × 8 years). The study was reviewed and approved by the Institutional Review Board (IRB) of Chungbuk National University (approval number CBIRB-202311-HR-024).

### 2.2. Data Acquisition

The main outcome, i.e., the number of snakebite cases by district and year, was obtained from two databases: the National Health Insurance Service (NHIS) and the National Emergency Department Information System (NEDIS). The NHIS reimburses hospitals for medical expenditures after reviewing medical practices in South Korea and manages the medical records of most Koreans, including diagnosed diseases, prescribed medications, basic individual information, and health examination data [11]. The NEDIS contains medical data from emergency medical institutions in South Korea, including diagnosed diseases with medical codes, basic patient information, and emergency locations [12]. From these databases, three outcome variables were obtained: the number of snakebite cases based on the residential locations of the patients (obtained from NHIS) and year (NHIS_R), the number of snakebite cases based on the locations of the emergency institutions (obtained from NEDIS) and year (NEDIS_I), and the number of snakebite cases based on the residential locations of the patients (obtained from NEDIS) and year (NEDIS_R).

The main explanatory variable was the level of deforestation by district and year. Hansen et al. developed fine-scale spatial data (approximately 30 m per pixel) on global forest cover change [13], which are publicly available as raster-type data [14]. The database includes forest cover data from 2000 and deforestation data from 2001 to 2023, with a resolution of approximately 30 m per pixel. The forest cover data have values ranging from 0 to 100, representing the percentage of forest cover, whereas the deforestation data have values from 0 to 23, representing the year of the deforestation event. The proportions of deforestation events for each district and year were obtained as the main explanatory variables, and the proportion of forest cover for each district and year was obtained as a covariate.

Additional covariates, including altitude, average temperature, population size, proportion of urban land cover, proportion of agricultural land cover, and level of ecological preservation, were collected. Altitude data for each district were obtained from Javis et al. [15], who provided spatial data at a 90 m resolution. The Automatic Synoptic Observation System (ASOS) provides yearly average temperature data for each monitoring site [16]. Point-level temperature data were interpolated to generate raster-type data covering the entire country, from which the annual mean temperature was extracted for each district. Although ASOS data include other meteorological variables such as precipitation and humidity, these were excluded from the study due to their high negative correlation with temperature. The population size for each district was obtained from the Korean Statistical Information Service [17]. The proportions of urban and agricultural land cover by district and year were acquired from the National Aeronautics and Space Administration’s Moderate Resolution Imaging Spectroradiometer [18]. The level of ecological preservation was obtained from the Environmental Geographic Information System [19]. The variables and their spatial and temporal resolutions are listed in Table 1.

### 2.3. Statistical Analysis

Descriptive analysis was performed to provide an overview of the spatial distribution of variables and the univariable differences between study units (district and year) with and without snakebite events. The spatial distribution was visualized using choropleth maps, and a Student’s *t*-test was employed to examine the univariable differences.

Twelve statistical models were employed and were categorized into four types (GLM_L, GLM_NB, CAR_L, and INLA_ NB) to assess the robustness of the associations between snakebites and deforestation. GLM_L and GLM_ NB represent ordinary logistic regression models and ordinary negative binomial regression models, respectively. CAR_L and INLA _NB represent conditional autoregressive (CAR) logistic models and negative binomial regression models using integrated nested Laplace approximation (INLA) method, respectively, which incorporate spatial autocorrelation. Logistic regression models included all study units to examine the effect of deforestation on the binary outcome (whether there is at least one snakebite case or not). On the other hand, study units that have zero case were excluded in the negative binomial models to assess the effect of deforestation on the number of snakebite cases (i.e., dose–response relationship). Odds ratios (ORs) and relative risks (RRs) were suggested for logistic and negative binomial regression models, respectively. The 95% confidence intervals (95% CI) and credible intervals (95% CrI) were presented for the GLM and spatial model (CAR and INLA) results, respectively. Each of the four categories included three models with different outcome variables (NHIS_R, NEDIS_I, and NEDIS_R for Models 1 to 3, respectively). A correlation matrix was estimated to examine the one-to-one association between the explanatory variables, and the variance inflation factor (VIF) was used to assess the multicollinearity of the models. R version 4.3.1. was used for all statistical analyses [20].

## 3. Results

**Figure 1:**
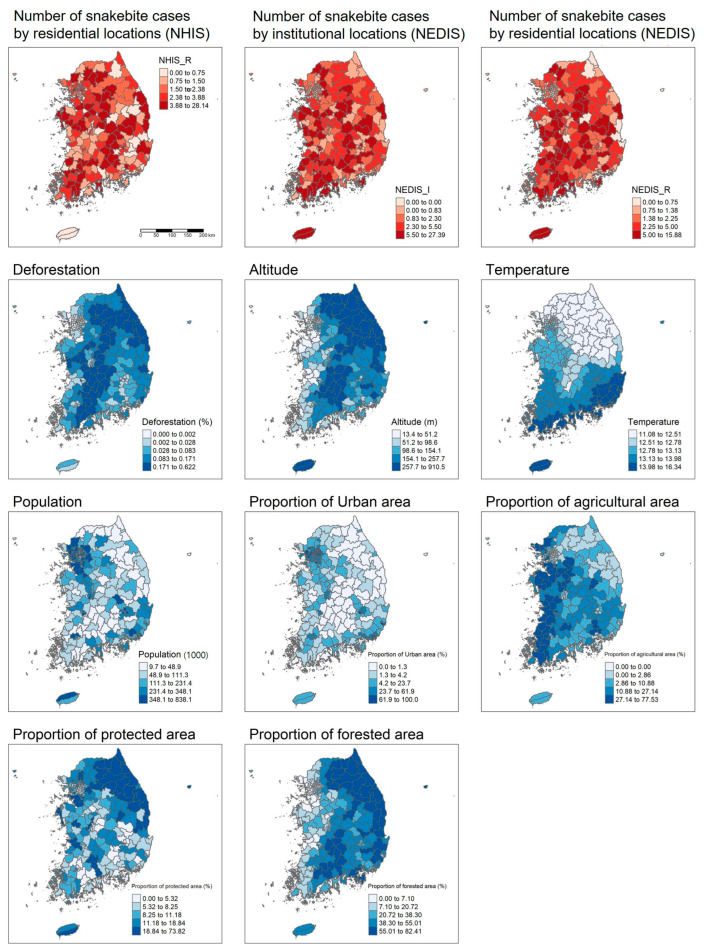
Spatial distribution of the explanatory (blue) and outcome (red) variables. Note: Outcome and explanatory variables (deforestation, altitude, temperature, population, proportion of urban area, proportion of agricultural area, proportion of protected area, and proportion of forested area) were extracted for 250 districts in South Korea. Although some of the variables (snake bite cases, deforestation, temperature, population size, urban and agricultural land cover) are time-varying, annual mean value was included in the figure.

Table 2 showed significant differences in several explanatory variables between study units with and without snakebites. Deforestation level, population size, and proportion of agricultural area proportion were generally higher in units experiencing snakebites. Conversely, altitude, annual mean temperature, and forest proportion were significantly lower in these units. No significant differences were observed in the proportions of urban and protected areas. Choropleth maps indicate similar spatial distributions for the three outcome variables, with spatial clustering evident for the eight covariates (Figure 1).

The associations between deforestation and snakebites were significantly positive across all 12 models (Table 3). ORs derived from logistic models (GLM_L 1-3 and CAR_L 1-3) ranged from 1.217 to 1.452, with higher ORs in GLM models compared with that in CAR models. The highest OR was observed in model 1 (OR and 95% CI: 1.452 and 1.256–1.691 for GLM_L1; 1.410 and 1.201–1.655 for CAR_L1). RRs estimated from negative binomial models (GLM_NB 1-3 and INLA_NB 1-3) ranged from 1.069 to 2.111, with the highest RR in model 1 for spatial model (RR and 95% CrI: 1.152 and 1.090–1.218) and model 3 for ordinary regression model (RR and 95% CI: 2.111 and 1.651–2.700). The absence of multicollinearity was confirmed by variance inflation factor (VIF) values < 4.

For covariates, altitude exhibited a significantly negative association in all models except GLM_L2 and CAR_L2, and population size showed a significantly positive association, except in the GLM_NB2 and GLM_NB3. The proportions of urban area and protected area demonstrated significant negative associations in models with an NEDIS outcome, whereas all models showed significant positive associations with forest proportion. (Supplementary Tables S1 and S2) Correlations between variables were suggested in Supplementary Figure S1.

## 4. Discussion

Our findings suggest a significant association between deforestation and increased snakebite incidence. Units with higher deforestation levels, larger population sizes, and greater agricultural area proportions consistently showed higher snakebite probabilities, with ORs between 1.217 and 1.452 and RRs between 1.078 and 1.175. Spatial analysis corroborated these results, indicating the clustering of snakebite incidences in areas with high deforestation and population densities.

The positive association between deforestation and the incidence of snakebites could be attributed to increased human–snake contact resulting from deforestation, and the associations could be found in other regions and contexts. An example of deforestation is the conversion of land for agricultural expansion. This process has led to increased migration of snakes into human settlements in search of food and shelter [21] and, thus, an increase in the incidence of snakebites. Deforestation can also affect zoonotic disease transmission [22]. For example, habitat destruction and deforestation are associated with outbreaks of diseases such as Ebola. In Africa, for instance, the destruction of bat habitats has increased human exposure to bats, which are natural reservoirs of the Ebola virus, leading to higher incidences of the disease in humans [23]. Furthermore, in Malaysia and Bangladesh, deforestation and agricultural expansion have increased contact between humans and fruit bats, which are the natural hosts of the Nipah virus, resulting in significant outbreaks [24]. Previous studies clearly show that conflicts between humans and wildlife are common [25]. One result of this conflict is zoonotic diseases, which spread more as human–wildlife interactions increase [26]. Snakebite incidences are similar. In epidemiology, spatial analysis plays an important role in preventing such dangers [27]. Figure 1 shows the distribution of snakebite incidences using data from NEDIS. However, expanded data collection and model compatibility are needed for improved analysis. Among the variables we studied, we created a map to show areas becoming more urbanized or used for agriculture. This map helps visualize snakebite distribution. Consistent with previous studies, we found positive associations between population size and agricultural land use.

Similar to previous studies, we found positive associations of snakebite incidence with population size and agricultural land use. An intuitive factor related to human–snake contact is population size. The association between population size and snake encounters is clear. When population size and snakebite incidence data for each district were combined using data from the Korean Statistical Information Service, we found significant statistical associations. As population size increases, so does the probability of encountering snakes, thereby raising the risk of snakebite incidences [28]. The risk increases due to the contact between humans and snakes, and it is also important to understand the patterns of snake activity [29]. Among the variables we studied, human density was considered, but mapping considering the distribution of snakes is also a role. Furthermore, we found a positive association between the proportion of agricultural land and snake encounters, consistent with findings in Malaysia and Bangladesh. In South Korea, where a significant portion of land is allocated for agriculture, we can find a positive association as encounters with snakes increase in areas where people are actively engaged in agricultural activities [30]. An increase in the incidence of snakebites in agricultural areas, while no such increase was observed in regions with a high proportion of forested land. This discrepancy appears to be attributable to differences in human activity patterns and the conditions under which snakes inhabit these environments. In cultivated lands, such as rice paddies and fields, snake habitats are more exposed. Additionally, snakes that prefer high-temperature and humid environments are more likely to be found in irrigation systems or control facilities designed to protect crops, leading to increased encounters with humans and a higher likelihood of snakebite incidences. Conversely, in areas with a high proportion of forested land, snakes have greater access to hiding spaces compared to agricultural fields, which reduces human contact and subsequently lowers the incidence of snakebites.

The associations of some factors are not considered in previous studies. In previous studies, it was found that snakebite incidents in Colombia increased along with rising precipitation levels. However, no significant association was identified between precipitation and snakebite incidents in South Korea. Additionally, earlier research indicated that snakebite occurrences were more frequent in areas with higher urbanization rates. However, our findings confirmed that there is no significant association between urbanization and snakebite accidents in the country. This suggests that urbanization can create environments less suitable for snakes by destroying their natural habitats and increasing human presence, thereby minimizing the risk [30]. Since climate change affects snake activity patterns and land uses [31], it is also necessary to constantly monitor various variables caused by climate change. We need to highlight the need for a more nuanced understanding of the relationships between snakebite incidence and environmental factors. The varying results may be influenced by differing regional characteristics. In addition, we explored the impact of other environmental variables that may explain increases in the incidence of snakebites. The incidence of snakebites was lower in first-grade ecological zones, which are well-preserved and may limit human activity and the possibility of human–snake encounters [29].

Our study has several limitations. First, we extracted data based on reported snakebite incidents at the administrative district level, which might not accurately reflect the actual incident locations. Second, while the analysis relied on population density, it is essential to clarify that a higher population density increases the likelihood of snake encounters. This does not necessarily imply that population growth directly correlates with snakebites. Instead, areas with greater transient populations, such as tourists or hikers, can lead to higher incident rates. Thus, data may be missing, especially in cases where an injured person was not a permanent resident of the district. To mitigate potential biases, we propose considering snake density rather than just population density. It will also play an important role in expanding and developing a spatial risk map in consideration of these various risk variables [32]. Third, we only derived data from emergency medical institutions that predominantly handle severe cases of snakebites. This may lead to an underestimation of the overall incidence of snakebites, as less severe cases are likely not recorded in emergency medical institutions. Fourth, the ORs suggested by logistic regression models could overestimate effect size. Fifth, the interpretation of our results regarding covariates should be cautious because our statistical models were designed to investigate the associations of deforestation. Different covariates should be included in further studies to explore the effect of covariates in this study.

## 5. Conclusions

In conclusion, we investigate how deforestation may influence snakebite risks in South Korea, where reliable healthcare data are available. Our study underscores the associations between environmental factors and snakebite incidents, emphasizing the need for region-specific regulations. Effective risk management should consider local ecology, land use, and population dynamics. In addition, this study presents a new approach for the prevention and management of snakebite accidents through the connection with the World Health Organization’s (WHO) strategy for preventing snakebites and South Korea’s community-based disaster management initiative [33]. The results show the potential to develop a map of snakebite accident risk over time and space. Such maps can provide better predictive performance by incorporating environmental factors such as forest-only events into machine learning models. Future research will require additional data collection and analysis to more clearly reveal the causal relationship associated with snakebite accidents. This will provide an effective response strategy that reflects both regional characteristics and global perspectives.

## Figures and Tables

**Table 1:** Explanatory variables employed in this study.

Variables	Type of Spatial Data	Temporal Resolution
Deforestation level (%)	Raster	Yearly
Altitude (m)	Raster	NA
Annual mean temperature (°C)	Point	Yearly
Population size (10^3^)	Non-spatial data	Yearly
Proportion of urban area (%)	Raster	Yearly
Proportion of agricultural area (%)	Raster	Yearly
Proportion of protected area (%)	Raster	NA
Proportion of forest (%)	Raster	NA (in 2000)

**Table 2:** Descriptive analysis for the explanatory variables.

Variables	Mean ± SD		*p* Value (*t*-Test)
	Study Units * with Snakebite Events (N = 1456)	Study Units * Without Snakebite Events (N = 544)	
Deforestation level (%)	0.10 ± 0.14	0.08 ± 0.11	0.002
Altitude (m)	165.89 ± 144.37	182.41 ± 161.08	0.036
Annual mean temperature (°C)	13.11 ± 0.95	13.41 ± 1.20	<0.001
Population size (10^3^)	220.63 ± 172.28	169.56 ± 133.14	<0.001
Proportion of urban area (%)	28.57 ± 33.38	27.00 ± 30.63	0.321
Proportion of agricultural area (%)	15.22 ± 18.82	12.11 ± 17.32	<0.001
Proportion of protected area (%)	13.82 ± 12.75	14.16 ± 13.77	0.613
Proportion of forest (%)	30.51 ± 23.43	33.98 ± 23.24	0.003

* Study units were each district and year.

**Table 3:** Associations between deforestation and snakebites in multiple statistical models.

Model Type	Outcome Variable	Ordinary Regression	Spatial Regression
Logistic regression	NHIS_R	1.452 (1.256–1.691)	1.410 (1.201–1.655)
Logistic regression	NEDIS_I	1.235 (1.089–1.406)	1.217 (1.055–1.400)
Logistic regression	NEDIS_R	1.233 (1.066–1.434)	1.232 (1.057–1.445)
Negative binomial model	NHIS_R	1.190 (1.131–1.253)	1.152 (1.090–1.218)
Negative binomial model	NEDIS_I	2.055 (1.294–3.265)	1.069 (1.002–1.140)
Negative binomial model	NEDIS_R	2.111 (1.651–2.700)	1.092 (1.037–1.150)

Note: The ordinary regression models were implemented by glm function in R. In terms of spatial regression, both conditional autoregressive model and integrated nested Laplace approximation were used for logistic regression and negative binomial models, respectively.

## Data Availability

The data presented in this study are available on request from the corresponding author. (The data are not publicly available due to privacy or ethical restrictions.).

## Supplementary Materials

The supplementary materials can be downloaded at: https://www.mdpi.com/article/10.3390/ani15020198/s1, Figure S1: Correlations between variables included in the models. Table S1: Association of deforestation on presence of snakebites; Table S2: Association of deforestation on the number of snakebites.
