# Supplementary material for: Association Between Deforestation and the Incidence of Snakebites in South Korea
Source: Animals (Basel). 2025 Jan 13;15(2):198. doi: 10.3390/ani15020198 (PMC11758619; doi:10.3390/ani15020198)
Supplement: Supplementary file 1 [file animals-15-00198-s001.zip › animals-3359566-supplementary.pdf]

Supplementary Materials

Supplementary Table S1. Association of deforestation on presence of snakebites

Outcome variable	Explanatory Variables	GLM (Odds ratio and 95% confidence intervals)	CAR (Odds ratio and 95% credible intervals)
NHIS_R	Deforestation level	1.452 (1.256 - 1.691)	1.410 (1.201 - 1.655)
	Altitude	0.617 (0.503 - 0.756)	0.624 (0.490 - 0.772)
	Annual mean temperature	0.740 (0.659 - 0.830)	0.732 (0.526 - 0.894)
	Population size	1.636 (1.414 - 1.900)	1.930 (1.593 - 2.448)
	Proportion of urban area	1.148 (0.958 - 1.377)	1.137 (0.934 - 1.422)
	Proportion of agricultural area	1.253 (1.080 - 1.458)	1.191 (0.963 - 1.427)
	Proportion of protected area	0.901 (0.803 - 1.012)	0.929 (0.818 - 1.065)
	Proportion of forest	1.732 (1.430 - 2.115)	1.812 (1.480 - 2.251)
	Year	1.026 (0.981 - 1.073)	1.028 (0.979 - 1.080)
NEDIS_I	Deforestation level	1.235 (1.089 - 1.406)	1.217 (1.055 - 1.400)
	Altitude	1.058 (0.874 - 1.279)	0.936 (0.757 - 1.164)
	Annual mean temperature	1.337 (1.189 - 1.506)	0.956 (0.765 - 1.172)
	Population size	1.962 (1.701 - 2.273)	2.179 (1.857 - 2.622)
	Proportion of urban area	0.434 (0.355 - 0.527)	0.472 (0.377 - 0.591)
	Proportion of agricultural area	1.422 (1.238 - 1.635)	1.257 (1.068 - 1.495)
	Proportion of protected area	0.878 (0.782 - 0.985)	0.873 (0.757 − 1.000)
	Proportion of forest	1.370 (1.158 - 1.628)	1.383 (1.151 - 1.665)
	Year	0.934 (0.894 - 0.974)	0.935 (0.892 - 0.980)
NEDIS_R	Deforestation level	1.233 (1.066 - 1.434)	1.232 (1.057 - 1.445)
	Altitude	0.990 (0.809 - 1.214)	0.963 (0.770 - 1.186)
	Annual mean temperature	1.122 (0.996 - 1.266)	1.040 (0.849 - 1.214)
	Population size	2.180 (1.866 - 2.562)	2.199 (1.879 - 2.597)
	Proportion of urban area	0.545 (0.452 - 0.654)	0.553 (0.458 - 0.675)
	Proportion of agricultural area	1.488 (1.266 - 1.757)	1.441 (1.184 - 1.723)
	Proportion of protected area	0.822 (0.736 - 0.918)	0.821 (0.730 - 0.921)
	Proportion of forest	1.328 (1.108 - 1.600)	1.323 (1.098 - 1.601)
	Year	0.944 (0.902 - 0.988)	0.945 (0.901 - 0.992)

Supplementary Table S2. Association of deforestation on the number of snakebites

Outcome variable	Explanatory Variables	GLM (Relative risk and 95% confidence intervals)	INLA (Relative risk and 95% credible intervals)
NHIS_R	Deforestation level	1.190 (1.131 - 1.253)	1.152 (1.090 -1.218)
	Altitude	0.809 (0.741 - 0.883)	0.778 (0.702 -0.863)
	Annual mean temperature	0.912 (0.863 - 0.963)	0.850 (0.761 -0.951)
	Population size	1.099 (1.043 - 1.158)	1.143 (1.081 -1.207)
	Proportion of urban area	0.935 (0.864 - 1.013)	0.969 (0.881 -1.065)
	Proportion of agricultural area	1.099 (1.034 - 1.167)	1.030 (0.960 -1.104)
	Proportion of protected area	0.929 (0.886 - 0.975)	0.975 (0.924 -1.029)
	Proportion of forest	1.348 (1.249 - 1.457)	1.355 (1.247 -1.473)
	Year	0.998 (0.980 - 1.016)	1.002 (0.984 -1.021)
NEDIS_I	Deforestation level	2.055 (1.294 - 3.265)	1.069 (1.002 -1.140)
	Altitude	0.351 (0.171 - 0.722)	0.872 (0.782 -0.971)
	Annual mean temperature	0.972 (0.617 - 1.531)	0.999 (0.916 -1.088)
	Population size	1.038 (0.638 - 1.688)	1.091 (1.016 -1.172)
	Proportion of urban area	0.346 (0.152 - 0.790)	0.761 (0.658 -0.880)
	Proportion of agricultural area	1.638 (0.952 - 2.817)	0.963 (0.882 -1.052)
	Proportion of protected area	0.349 (0.206 - 0.593)	0.841 (0.770 -0.918)
	Proportion of forest	6.081 (3.165 - 11.681)	1.361 (1.235 -1.500)
	Year	0.716 (0.605 - 0.848)	0.952 (0.931 -0.973)

NEDIS_R	Deforestation level	2.111 (1.651 - 2.700)	1.092 (1.037 -1.150)
	Altitude	0.385 (0.260 - 0.570)	0.865 (0.790 -0.948)
	Annual mean temperature	1.236 (0.978 - 1.564)	1.051 (0.979 -1.129)
	Population size	1.128 (0.873 - 1.456)	1.136 (1.073 -1.203)
	Proportion of urban area	0.415 (0.280 - 0.615)	0.743 (0.670 -0.823)
	Proportion of agricultural area	1.271 (0.961 - 1.681)	1.000 (0.934 -1.070)
	Proportion of protected area	0.564 (0.440 - 0.723)	0.880 (0.827 -0.936)
	Proportion of forest	3.845 (2.687 - 5.503)	1.354 (1.252 -1.465)
	Year	0.801 (0.735 - 0.872)	0.953 (0.937 -0.970)

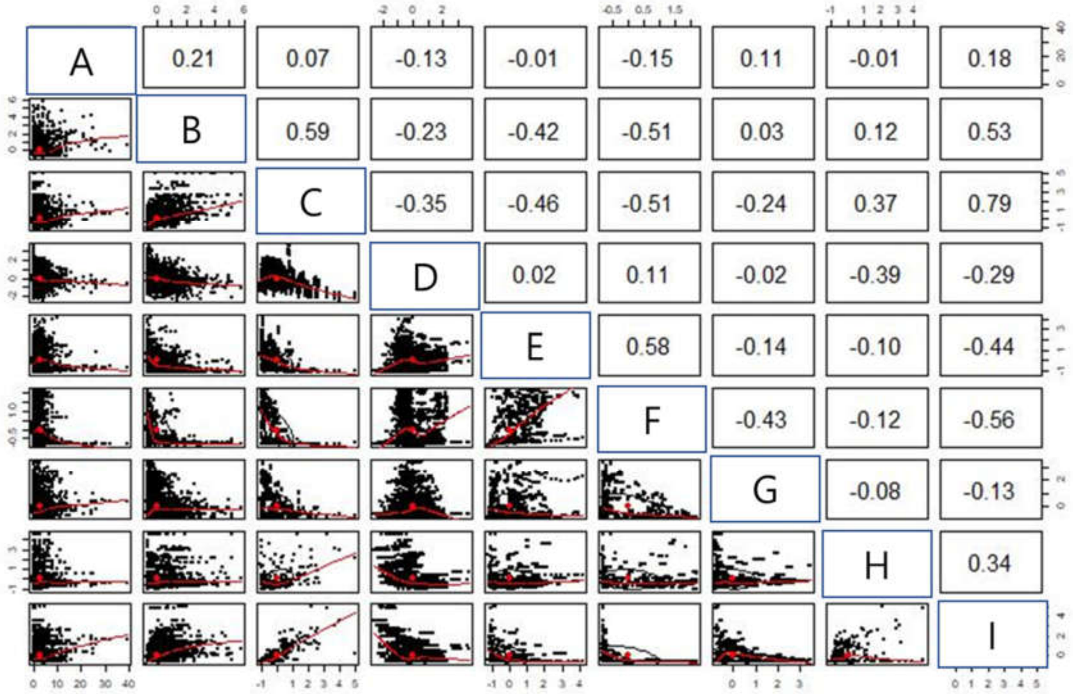

Supplementary Figure S1. Correlations between variables included in the models

Note: A indicates snakebite cases (NHIS), B indicates deforestation, C indicates altitude, D indicates temperature, E indicates population, F indicates urban land use, G indicate agriculture land use, H indicates protected area, I indicates treecover land use.
